# Repeated Measures Correlation

**DOI:** 10.3389/fpsyg.2017.00456

**Published:** 2017-04-07

**Authors:** Jonathan Z. Bakdash, Laura R. Marusich

**Affiliations:** ^1^US Army Research Laboratory, Human Research and Engineering Directorate Aberdeen Proving Ground, USA; ^2^US Army Laboratory South Field Element, Human Research and Engineering Directorate, University of Texas Arlington Arlington, TX, USA

**Keywords:** correlation, repeated measures, individual differences, intra-individual, statistical power, multilevel modeling

## Abstract

Repeated measures correlation (rmcorr) is a statistical technique for determining the common within-individual association for paired measures assessed on two or more occasions for multiple individuals. Simple regression/correlation is often applied to non-independent observations or aggregated data; this may produce biased, specious results due to violation of independence and/or differing patterns between-participants versus within-participants. Unlike simple regression/correlation, rmcorr does not violate the assumption of independence of observations. Also, rmcorr tends to have much greater statistical power because neither averaging nor aggregation is necessary for an intra-individual research question. Rmcorr estimates the common regression slope, the association shared among individuals. To make rmcorr accessible, we provide background information for its assumptions and equations, visualization, power, and tradeoffs with rmcorr compared to multilevel modeling. We introduce the R package (rmcorr) and demonstrate its use for inferential statistics and visualization with two example datasets. The examples are used to illustrate research questions at different levels of analysis, intra-individual, and inter-individual. Rmcorr is well-suited for research questions regarding the common linear association in paired repeated measures data. All results are fully reproducible.

## Introduction

Correlation is a popular measure to quantify the association between two variables. However, widely used techniques for correlation, such as simple (ordinary least squares with a single independent variable) regression/Pearson correlation, assume independence of error between observations (Howell, 1997; Johnston and DiNardo, 1997; Cohen et al., 2003). This assumption does not pose a problem if each participant or independent observation is a single data point of paired measures (i.e., two data points corresponding to the same individual such as height and weight). For example, when correlating the current height and weight of people drawn from a random sample, there is no reason to expect a violation of independence.

However, the assumption of independence is violated in repeated measures, in which each participant provides more than one data point. For example, if a study collected height and weight for a sample of people at three time points, there would likely be non-independence in the errors of the three observations belonging to the same person. Analyzing non-independent data with techniques that assume independence is a widespread practice but one that often produces erroneous results (Kenny and Judd, 1986; Molenaar, 2004; Aarts et al., 2014). One common solution is to average the repeated measures data for each participant prior to performing the correlation. This aggregation may resolve the issue of non-independence but can produce misleading results if there are meaningful individual differences (Estes, 1956; Myung et al., 2000). Furthermore, analysis of individual differences can be useful as a strong test for theory (Underwood, 1975; Vogel and Awh, 2008).

Bland and Altman (1995a,b) introduced the within-participants correlation in biostatistics to analyze the common intra-individual association for paired repeated measures, which are two corresponding measures assessed for each participant/case/individual on two or more occasions. Here, we refer to the technique as the repeated measures correlation (rmcorr). Rmcorr accounts for non-independence among observations using analysis of covariance (ANCOVA) to statistically adjust for inter-individual variability. By removing measured variance between-participants, rmcorr provides the best linear fit for each participant using parallel regression lines (the same slope) with varying intercepts. Like a Pearson correlation coefficient (r), the rmcorr coefficient (*r*_rm_) is bounded by −1 to 1 and represents the strength of the linear association between two variables. Also akin to the Pearson correlation, the null hypothesis for rmcorr is ρ_*rm*_ = 0, and the research/alternative hypothesis is ρ_rm_ ≠ 0. Unlike the Pearson correlation, which assesses the inter-individual association because it assumes each paired data point is Independent and Identically Distributed (IID), rmcorr evaluates the overall or common intra-individual association between two measures. Because rmcorr takes into account non-independence, it tends to yield much greater power than data that are averaged in order to meet the IID assumption for simple regression/correlation. Hence, rmcorr can detect associations between variables that might otherwise be obscured or spurious due to aggregation or treating non-independent values as IID.

Conceptually, rmcorr is close to a null multilevel model (i.e., varying intercept and a common slope for each individual), but the techniques differ on how they treat/pool variance. Rmcorr assesses the common intra-individual variance in data, whereas multilevel modeling can simultaneously analyze different sources of variance using fixed and random effects. The tradeoff with more complex multilevel models is that they require more data and are more challenging to specify and interpret than simpler analysis of variance (ANOVA)/regression models, such as rmcorr. However, the flexibility of multilevel modeling has benefits: Overall and individual differences can be analyzed simultaneously, models of varying complexity can be systematically compared, and they can provide greater insights into individual differences.

Besides multilevel modeling, we contend there are no other widely used techniques that can correctly model paired and repeated measures data that are continuous. The common correlation techniques (e.g., Pearson, Kendall, and Spearman) for paired data and canonical correlation for multivariate data all assume independent observations. Repeated observations can be modeled with multivariate analysis of variance (MANOVA) and repeated measures ANOVA, but they are for factorial designs and not paired data. While ANCOVA can operate on paired data, its purpose (to statistically adjust for a nuisance, within-participants variance, in each individual) is opposite to that of rmcorr (using one of the paired measures to statistically adjust for between-participants variance) (see Rmcorr and ANCOVA for details).

Despite the potential utility of rmcorr for repeated measures data, it is relatively unknown in psychological research. To address this gap, the paper is structured as follows. The background describes how rmcorr works, its relation to ANCOVA, and the tradeoffs for rmcorr compared to multilevel modeling. Next, we provide an overview of the **rmcorr R package** using two examples with real data. Last, we summarize when rmcorr may be informative and potential applications.

All graphs and results are fully reproducible using R (R Core Team, 2017), the **rmcorr R package** https://cran.r-project.org/web/packages/rmcorr/, and the accompanying R Markdown document: https://osf.io/djphm/. R packages used in the paper, but not cited in the references, are listed in Appendix A.

## Background

To convey a conceptual understanding of rmcorr, we first provide visualizations comparing rmcorr and simple regression/correlation using hypothetical data. Then, to explain the underlying mechanics of rmcorr we provide an overview of ANCOVA for aspects relevant to rmcorr; key assumptions (e.g., parallel slopes); and the notation, data structure, and formulas for rmcorr (equations for calculations and degrees of freedom). Last, we calculate power curves for rmcorr to show the benefits of repeated measures for higher statistical power relative to simple regression/correlation.

### Visualization: rmcorr plot

**Figure 1:**
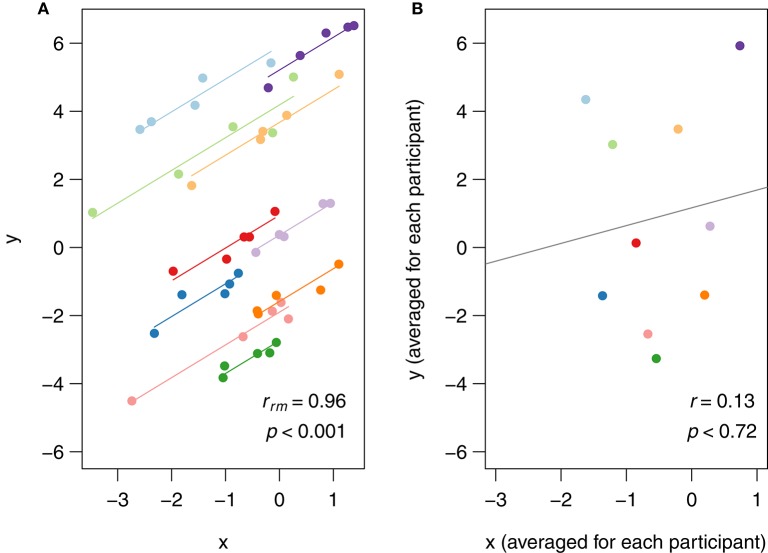
**(A)** Rmcorr plot: rmcorr plot for a set of hypothetical data and **(B)** simple regression plot: the corresponding regression plot for the same data averaged by participant.

In rmcorr, separate parallel lines are fit to the data from each participant. The sign of the rmcorr coefficient (i.e., positive or negative) is indicated by the direction of the common regression slope. The left panel of Figure 1 shows an rmcorr plot for a set of hypothetical repeated measures data, with 10 participants providing five data points each. Each participant's data and corresponding line are shown in a different color. The computed rmcorr value for this notional data is 0.96. The right panel shows the same notional data, but with each subject's data averaged into one data point each. The regression line is plotted with this averaged data. Note that the computed correlation coefficient for this averaged data is much smaller (0.13) and is not significant. In this example, rmcorr captures the strong intra-individual relationship between the two variables that is missed by using averaged data.

#### Interpreting results

**Figure 2:**
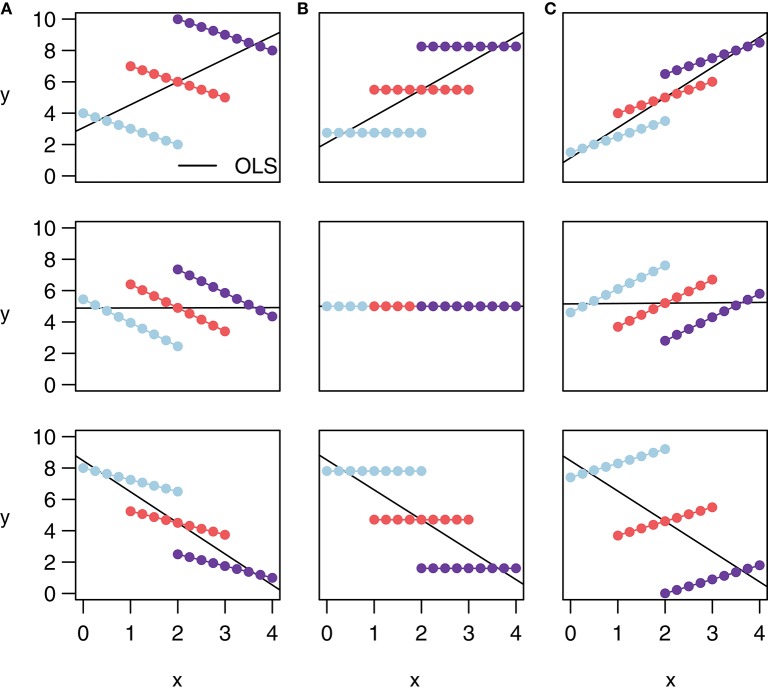
**These notional plots illustrate the range of potential similarities and differences in the intra-individual association assessed by rmcorr and the inter-individual association assessed by ordinary least squares (OLS) regression**. Rmcorr-values depend only on the intra-individual association between variables and will be the same across different patterns of inter-individual variability. **(A)** *r*_*rm*_ = −1: depicts notional data with a perfect negative intra-individual association between variables, **(B)** *r*_*rm*_ = 0: depicts data with no intra-individual association, and **(C)** *r*_*rm*_ = 1: depicts data with a perfect positive intra-individual association. In each column, the relationship *between* subjects (inter-individual variability) is different, which does not change the rmcorr-values within a column. However, this does change the association that would be predicted by OLS regression (black lines) if the data were treated as IID or averaged by participant.

Note that rmcorr can reveal very different within-participant associations among similar patterns of aggregated data, as depicted with notional data in Figure 2. All the data in a given row exhibit the same relationship when treated (incorrectly) as IID, indicated by the black simple regression line in each cell. However, across columns the intra-individual association is quite different. This phenomenon is why generating an rmcorr plot can be helpful for understanding a given dataset. As with other statistical techniques, visualization is key for interpreting results (Tukey, 1977).

Figure 2 also depicts examples of Simpson's Paradox (note in particular Panel (A), Row 1, and Panel (C), Row 3), in which patterns at a higher level of analysis (e.g., sample, experiment, study, or aggregated data) conflict with patterns at a lower level of analysis (Tu et al., 2008; Robinson, 2009; see Kievit et al., 2013; e.g., individual). For patterns at one level of analysis to generalize to another, the data must be ergodic between levels (Molenaar, 2004; Molenaar and Campbell, 2009). Rmcorr, and especially the rmcorr plot, may be useful for understanding non-ergodic data that have intra-individual and inter-individual patterns that do not generalize to each other.

**Figure 3:**
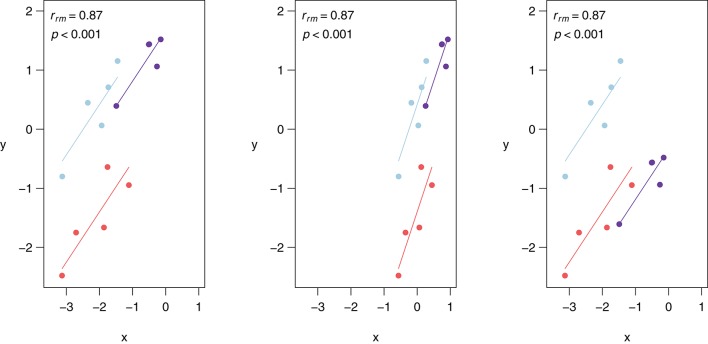
**Rmcorr-values (and corresponding *p*-values) do not change with linear transformations of the data, illustrated here with three examples: (A)** original, **(B)** *x/*2 + 1, and **(C)** *y* − 1.

Similar to Pearson correlation, linear transformations (i.e., addition, subtraction, multiplication, and/or division) of data do not alter the rmcorr value because the relationships among variables are preserved. More specifically, a linear transformation can be applied to the entire dataset, all data for one or more participants, or even by applying different transformations to the data of each participant without affecting the value of rmcorr. Figure 3 depicts linear transformations for hypothetical data in which effect sizes do not change. The first panel shows the rmcorr plot for a set of three participants, with five data points each. The second panel shows the resulting rmcorr plot when the x-variable values for all participants are transformed by dividing by 2 and adding 1. The third panel depicts the rmcorr plot when the y-variable values for only one subject are transformed by subtracting 2. Note that the rmcorr values are the same for the original data and the two transformations.

### Rmcorr and ANCOVA

Rmcorr is calculated using a form of ANCOVA, thus the two techniques share assumptions and equations (Howell, 1997; Miller and Chapman, 2001; Tabachnick and Fidell, 2007). However, rmcorr is an atypical application of ANCOVA. Typically, ANCOVA is used to determine the effect of a categorical independent variable upon a continuous dependent variable by removing the observed variance of a second “nuisance” continuous variable, or covariate (Howell, 1997; Miller and Chapman, 2001; Tabachnick and Fidell, 2007). Rmcorr, however, is used to determine the relationship between the two continuous variables, while controlling for the effect of the categorical variable, which in this case is the between-participants variance. In other words, the typical use of ANCOVA is opposite to the purpose of rmcorr.

Rmcorr is estimated using ANCOVA, albeit with an unusual model specification. ANCOVA is typically used to assess the effects of different (treatment or factor) levels upon a dependent measure, while controlling for the effects of another continuous variable (the covariate). For rmcorr, the participant is the factor level and the covariate is the second measure. We describe estimation of rmcorr by first providing the equation for a one-way ANCOVA (Equation 1); second, modifying this equation for rmcorr (Equation 2); and third, simplifying it (Equation 3). Last, we show the rmcorr table and calculations for the rmcorr coefficient (the direction is based on the sign of the slope).

#### Assumptions

The standard assumptions required for rmcorr include the standard ones for General Linear Model (GLM) techniques (e.g., Gelman and Hill, 2007) with a single exception: Independence of errors is relaxed in rmcorr. Major GLM assumptions include linearity (predictors are a linear function of the dependent measure), errors are IID [independent and identically distributed (i.e., equal variance)], and errors are normally distributed. Severe violations of the above assumptions could result in a biased model, which may be misleading or even uninterpretable.

In addition to the basic GLM assumptions, an additional assumption for ANCOVA is that the slopes indicating the relationship between the dependent variable and the covariate be parallel across conditions (e.g., Howell, 1997; Miller and Chapman, 2001; Tabachnick and Fidell, 2007)^1^. In practice, this assumption is considered to be met when there is no evidence of strong heterogeneity of slopes^2^. However, parallel lines are not an assumption for rmcorr; rather, rmcorr specifically tests for such a common association between variables. Therefore, the degree to which each subject's data is reflected by the common slope of the best-fit parallel lines is appropriately represented in the rmcorr effect size. When the relationship between variables varies widely across subjects, the rmcorr effect size will be near zero with confidence intervals also around zero. When there is no strong heterogeneity across subjects and parallel lines provide a good fit, the rmcorr effect size will be large, with tight confidence intervals.

If modeling varying slopes is important and there is sufficient data, the best approach would be fitting and comparing multilevel models (see Multilevel Modeling). Small effect sizes for rmcorr may be caused not only by heterogeneous slopes (poor model fit), however, but also by consistently near-zero slopes across subjects (see Interpreting Results and Figure 2B), or by restriction in the range of one or both measures (Cohen et al., 2003). Visualization of the data is critical to determine which of these is the underlying cause of a small effect size.

Two additional ANCOVA assumptions that are directly relevant to rmcorr are a linear association (linearity is also a standard GLM assumption) and high reliability for the covariate/measure (Howell, 1997; Miller and Chapman, 2001; Tabachnick and Fidell, 2007). A clear nonlinear association should be visually apparent from plotting the raw data and examining the rmcorr plot. One option is to apply a transformation to the data to make the association more linear (e.g., Cohen et al., 2003). Another possibility is to fit a nonlinear multilevel model. There are many methods for assessing reliability (consistency) (e.g., John and Benet-Martinez, 2014). Reliability is a complicated topic that is beyond the scope of this paper. However, if measurement reliability is previously known, or can be calculated, a correction for attenuation (e.g., John and Benet-Martinez, 2014) could be applied to the rmcorr coefficient.

#### Rmcorr notation and data format

The notation for rmcorr is defined in Table 1, and the data format for the rmcorr and Pearson correlation are shown in Table 2.

**Table 1:** **Notation**.

**Notation**	**Definition**
*L*	Total number of paired repeated measures (number of rows)
*N*	Sample size (number of unique individuals)
*k* = $\frac{L}{N\;}$	Mean number of paired repeated measures per individual

*The notation and their definitions. Rmcorr can accommodate unbalanced designs (i.e., differing numbers of paired repeated measures per participant)*.

**Table 2:** **Data Format**.

**Participant (i)**	**Trial (j)**	***X***	***Y***
**(A) Rmcorr DATA FORMAT, *k* = 3**.			
1	1		
1	2		
1	3		
2	1		
2	2		
2	3		
…	…		
…	…		
…	…		
N	1		
N	2		
N	3		
**Participant**	***X***		***Y***
**(B) SIMPLE REGRESSION/CORRELATION**.			
1			
2			
…			
N			

*The two measures are represented by X and Y. Lines between participant rows indicate independence between observations. Data for rmcorr is in a wide/long format with a minimum of two repeated observations. The i subscript represents each participant and the j subscript indicates each trial (per participant)*.

Rmcorr data is in a long or narrow format with separate columns for the participant and paired measures, and separate rows for each repeated observation, labeled by participant (Table 2A). In contrast, each row of data formatted for the simple regression/correlation is presumed to be an independent observation (Table 2B). The distinction between the two data formats is similar to the difference between the person period format and the person level format used in longitudinal data analysis.

#### Equations and rmcorr table

Rmcorr is estimated using ANCOVA, albeit with an unusual model specification. ANCOVA is typically used to assess the effects of different (treatment or factor) levels upon a dependent measure, while controlling for the effects of another continuous variable (the covariate). For rmcorr, the participant is the factor level and the covariate is the second measure. We describe estimation of rmcorr by first providing the equation for a one-way ANCOVA (Equation 1); second, modifying this equation for rmcorr (Equation 2); and third, simplifying it (Equation 3). Last, we show the rmcorr table and calculations for the rmcorr coefficient (the direction is based on the sign of the slope).

The equation for a one-way ANCOVA with *i* participants and *j* (factor) levels (Howell, 1997; Tabachnick and Fidell, 2007) is:

(1) $$Y_{ij}\;=\;\mu +\;\tau_{j}\;+\;c\;+\;\varepsilon_{ij}$$

*Y*_*ij*_ is the dependent measure for the *i*th participant at the *j*th factor level.

μ is the overall mean.

τ_*j*_ is the effect of the *j*th factor level.

*c* is the covariate: $c\;=\;\beta \left(X_{ij}\;-\;\bar{X_{j}}\right)$ (β is overall slope coefficient for the covariate, *X*_*ij*_ is the value of the covariate for the *i*th participant at the *j*th factor level, and $\bar{X_{j}}$ is the mean of the covariate values at the *j*th factor level).

ε_*ij*_ is the error for the *i*th participant at the *j*th factor level (the error is the difference between the actual value of dependent measure and its estimated value, for the *i*th participant at the *j*th factor level).

In Equations 2 and 3, Equation 1 is rewritten for rmcorr to show one measure as a function of its mean value, participant, and the covaried value of the other measure. Note following Equation 1, *i* and *j* are now exchanged for consistency: *j* = participant and *i* = trial or repeated measure.

(2) $$\begin{array}{rcl} {Measure1}_{ij}=\;{\bar{Measure1}}_{j}+\;Participant_{j} & & +\;\beta \left({Measure2}_{ij}-\;{\bar{Measure2}}_{j}\right)+\;\varepsilon_{ij} \end{array}$$

*Measure*1 and *Measure*2 are exchangeable.

*Measure*1_*ij*_ is the value of *Measure*1 for the *j*th participant at their *i*th trial.

${\bar{Measure1}}_{j}$ is the mean of *Measure*1 (all *i* trials) for the *j*th participant.

*Participant*_*j*_ is a unique identifier that acts as a dummy or proxy coded variable.

β is the value of the covariate, which is the overall or common slope.

*Measure*2_*ij*_ is the value of *Measure*2 for the *j*th participant at their *i*th trial.

${\bar{Measure2}}_{j}$ is the mean of *Measure*2 (all *i* trials) for the *j*th participant.

ε_*ij*_ is the error for the *j*th participant at their *i*th trial.

Equation 2 is rewritten to calculate the predicted value of the rmcorr regression line for each participant by trial. We drop the error term because we do not fit a confidence interval for the regression line.

(3) $$\begin{array}{r} {Measure1}_{ij}^{\prime }=\;{\bar{Measure1}}_{j}+\;Participant_{j}\\ +\;\beta \left({Measure2}_{ij}-\;{\bar{Measure2}}_{j}\right) \end{array}$$

$Measure1_{ij}{}^{\prime }$ is the predicted y-value of *Measure*1 for the *j*th participant at their *i*th trial.

*Measure*2_*ij*_ is the actual x-value which corresponds to the predicted y-value in the regression line.

Please note that the **rmcorr package** has always produced the corrected results.

Like a regression or ANOVA table, the rmcorr table summarizes quantitative results (Table 3).

**Table 3:** **Rmcorr Table**.

**Source**	**Degrees of Freedom**	**Sum of Squares**	**Mean Square Error**	***F* ratio**	***p*-value**
Participants	*N –* 1	*SS*_*Participant*_	$\frac{SS_{Participant}}{N-1}$	$\frac{MSE_{Measure}}{MSE_{Error}}$	
Measure	1	*SS*_*Measure*_	$\frac{SS_{Measure}}{1}$	$\frac{MSE_{Measure}}{MSE_{Error}}$	Significance value is determined by the *F*-ratio: *F*(*Measure df* (1), *Error df*)
Error	*N(k − 1)* − 1	*SS*_*Error*_	$\frac{SS_{Error}}{N\left(k-1\right)-1}$		
Total	*(Nk) − 1*	*SS*_*Total*_	$\frac{SS_{Total}}{\left(Nk\right)-1}$		

*This table is similar to a regression/ANOVA table*.

Based on the sums of squares values for the measure and error, the rmcorr correlation coefficient is calculated as follows:

(4) $$\begin{array}{l} \;r_{rm}\;=\;\sqrt{\frac{SS_{Measure}}{SS_{Measure}\;+\;SS_{Error}}}\\ \text{Sign of }r_{rm}\;\left(\text{positive or negative}\right)=\text{ Sign of }\beta \end{array}$$

Whether *r*_*rm*_ takes a positive or negative value is based on the sign of β (the common slope), in Equation 3. Additionally, the sign of the slope should be apparent in the rmcorr plot.

It does not matter which of the two measures is specified as the dependent variable and which one is the covariate. This is equivalent to switching the dependent and independent variable in simple regression/correlation. In rmcorr, the variable specification only changes the values of the sums of squares. All other parameter estimates are unchanged.

We recommend reporting rmcorr descriptively with the rmcorr plot and quantitatively using the *r*_*rm*_ (error degrees of freedom in parentheses), *p*-value, and a 95% confidence interval for *r*_*rm*_ (see **rmcorr R package** section). Presenting the point (*r*_*rm*_) and interval estimate (95% CI) of effect sizes is a meta-analytic approach (Wilkinson, 1999; Cumming, 2014), and is consistent with current statistical best practices.

### Degrees of freedom and power

Because rmcorr uses repeated measures, it will generally have higher degrees of freedom and power than a simple regression/correlation with averaged data. The covariate in rmcorr slightly reduces the degrees of freedom, by one, but this loss is miniscule compared to the gains because of repeated measures. Consequently, rmcorr generally has much higher power than Pearson correlation with averaged data.

#### Degrees of freedom

To calculate statistical power for rmcorr, we provide the exact degrees of freedom as well as approximations for convenience. The exact degrees of for rmcorr (from Table 3) are:

(5) N(k−1) −1

Where *k* is the (average) number of repeated measures per participant and *N* is the total number of participants. Note the loss of a degree of freedom for the covariate. The degrees of freedom for rmcorr can be approximated as a multiplier of (*k* − 1) times the degrees of freedom for the Pearson correlation (*N* − 2). See Appendix B for the proof for this approximation.

In standard power tables or programs such as G^*^Power (Faul et al., 2009), users may calculate power for rmcorr by using the entries for a Pearson correlation, but substituting in the appropriate degrees of freedom for rmcorr.

#### Power

**Figure 4:**
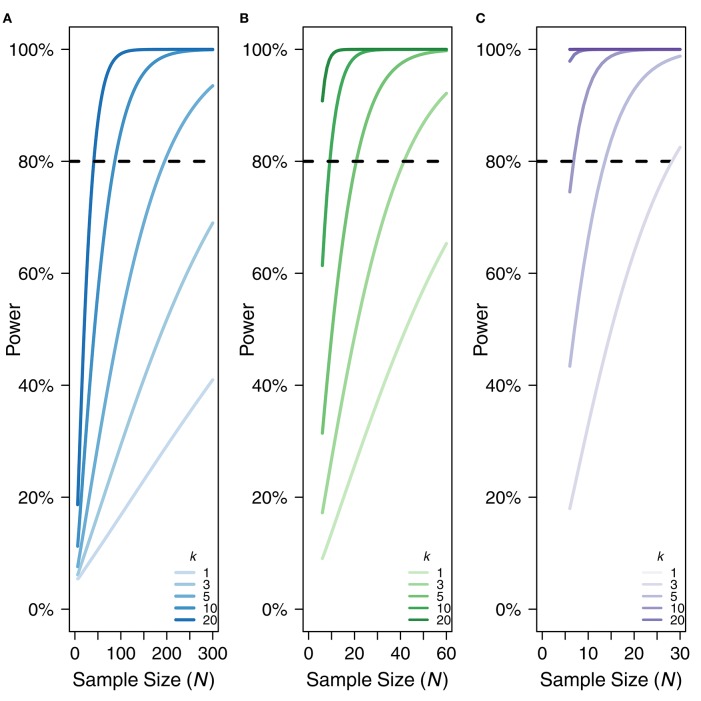
**Power curves for (A)** small, *r*_*rm*_, and *r* = 0.10, **(B)** medium, *r*_*rm*_, and *r* = 0.3, and **(C)** large effect sizes, *r*_*rm*_, and *r* = 0.50. X-axis is sample size. Note the sample size range differs among the panels. Y-axis is power. *k* denotes the number of repeated paired measures. Eighty percent power is indicated by the dotted black line. For rmcorr, the power of *k* = 2 is asymptotically equivalent to *k* = 1. A comparison to the power for a Pearson correlation with one data point per participant (*k* = 1) is also shown.

Because rmcorr is able to take advantage of multiple data points per participant, it generally has much greater statistical power than a standard Pearson correlation using averaged data. Low power typically overestimates effect sizes (e.g., Button et al., 2013). Power for rmcorr increases exponentially when either the value of *k* (the number of repeated observations) or the value of *N* (the total number of unique participants) increases. Figure 4 illustrates the power curves over different values of ***k*** and ***N*** for small, medium, and large effect sizes.

### Multilevel modeling

A powerful and flexible method for handling different sources of variance simultaneously is multilevel (linear) modeling^3^ (Kreft and de Leeuw, 1998; Singer and Willett, 2003; Gelman and Hill, 2007; see Aarts et al., 2014). Rmcorr can be viewed as a “light” version of multilevel modeling because it is comparable to a simple, null multilevel model with random/varying effects of intercept for each individual and a fixed effect (i.e., common/overall) slope (see Appendix C for direct comparisons). However, rmcorr only analyzes intra-individual variance. Multilevel modeling can simultaneously analyze both intra- and inter-individual variance using partial pooling, which permits varying slopes and other parameters that cannot be estimated with simpler techniques.

Compared to other types of pooling, and thus other statistical techniques, multilevel modeling has the unique advantage of being able to estimate variance at multiple hierarchical levels of analysis *simultaneously* using partial pooling^4^. Partial pooling estimates parameters at multiple levels by treating a lower level of analysis (e.g., individuals) as random/varying effects from a probability distribution drawn from a higher level of analysis (e.g., experiment) (see Gelman, 2005). Estimating random or varying effects requires sufficient, but not excessive, variation, and typically five or more levels (Bolker, 2015). Consequently, multilevel models with varying slopes will generally need more data than is required for rmcorr and other ANOVA techniques.

With partial pooling, multilevel models have the potential to provide far greater insight into individual differences and other patterns compared to ANOVA techniques. The main advantages of multilevel modeling are that it can accommodate much more complex designs than ANOVAs, such as varying slopes, crossed and nested factors—up to three hierarchical levels—and missing data. This flexibility may make it challenging to implement and understand compared to ANOVA (Gueorguieva and Krystal, 2004; Quené and van den Bergh, 2004). With more complex multilevel models, there is potential for overfitting or overparameterization (i.e., excessive free parameters given the amount of data and the model form). Overfitting may produce uninterpretable results, so model comparison is essential (Singer and Willett, 2003; Bates et al., 2015). However, concerns about model overfitting are general and extend to ANOVA/regression/correlation and numerous other techniques (Babyak, 2004; e.g., Aarts et al., 2014). Nevertheless, multilevel modeling can provide insights that are otherwise impossible with ANOVA/regression.

## Rmcorr R package

To make rmcorr more accessible to researchers, we have developed the **rmcorr package** for use in R (R Core Team, 2017). The package contains functions for both computing the rmcorr coefficient (as well as confidence intervals, etc.) and generating rmcorr plots. It also includes several example data sets, two of which are described in detail below. This package can be accessed in CRAN R: https://cran.r-project.org/web/packages/rmcorr/ and installed and loaded in R using the following commands:

1. install.packages(“rmcorr”)
2. library(rmcorr)

### Package overview

#### Package

The **rmcorr package** has two primary functions: *rmcorr* and *plot.rmc*.

1. *rmcorr:* This function takes as input repeated measures paired data and computes the repeated measures correlation coefficient. It takes the form:
rmc.out <- rmcorr(participant,
measure1, measure2, dataset, CIs =
c(“analytic,” “bootstrap”), nreps = 100,
bstrap.out = F)
  Where participant, measure1, and measure2 are variables giving the participant ID/number, observations for the first measure, and observations for the second measure, respectively, and dataset is a data frame containing these three variables. The function returns an rmc object, a list containing four primary components: The value of the rmcorr coefficient, error numerator degrees of freedom, the 95% confidence interval for the rmcorr coefficient, and the *p*-value for the rmcorr coefficient.
  An additional optional parameter, CIs, allows the user to specify if the confidence intervals generated by the function are computed analytically using the Fisher transformation or using a bootstrapping procedure. If bootstrapped confidence intervals are chosen, additional arguments specify the number of resamples and whether the function output will include the resampled rmcorr values.
2. *plot.rmc:* This function takes as input an rmc object (the output from the *rmcorr* function) and the dataset used to generate it. It produces a scatterplot of the repeated measures paired data, with each participant's data plotted in a different color. The function takes the form:
plot(rmc, dataset, overall = T, palette
= NULL, xlab = NULL, ylab = NULL,
overall.col = “gray60,” overall.lwd = 3,
overall.lty = 2,…)
  The overall parameter specifies whether a line should be plotted, indicating the regression line that would result from treating the data as independent observations (ignoring the repeated measures nature of the data). overall.col, overall.lwd, and overall.lty are optional parameters specifying the appearance of this line. The palette parameter allows the user to optionally choose a color palette for the plot. xlab and ylab are parameters for labeling the x- and y-axis, defaulting to the variable names in dataset. Finally, additional arguments to the generic plot function (…) can specify other aspects of the plot's appearance.

Help and examples for each of these functions can be accessed within R by typing help(function.name) or ?function.name. The **Rmcorr package** also includes three built-in example datasets: bland1995 the data described in Bland and Altman (1995a), raz2005 (the dataset used in the first example below), and gilden2010 (the dataset used in the second example below). More information about each dataset is accessible within R with the commands of help() or ?. In the sections below, we describe the bootstrapping procedure available in this package in more detail, and then provide examples of the package functions using real data.

#### Bootstrapping

The rmcorr effect size is estimated using a parametric confidence interval, which assumes normality but can be more robustly determined using bootstrapping. Bootstrapping does not require distributional assumptions and uses random resampling to estimate parameter accuracy (Efron and Tibshirani, 1994). The bootstrap for rmcorr is implemented by randomly drawing observations with replacement, within-individuals. This procedure is repeated on each individual, yielding a bootstrapped sample. The number of bootstrapped samples can be specified. Each bootstrap sample is then analyzed with rmcorr, producing a distribution of *r*_*rm*_ values. Last, these values are used to calculate the bootstrapped rmcorr coefficient (_*r*_*rm*_*boot*_) and its corresponding confidence interval (*CI*_*boot*_). There are a variety of methods for calculating a bootstrapped confidence interval (see (DiCiccio and Efron, 1996; Canty and Support, 2015). An example is presented in the documentation for the **rmcorr** package.

### Two example datasets

Two example datasets are shown using the **rmcorr** package to calculate inferential statistics and visualize results. These examples demonstrate the potential application of rmcorr for intra-individual research questions, and illustrate how and why results can differ from simple regression/correlation, which addresses inter-individual research questions. The first dataset is composed of repeated measures of age and brain structure volume over two time periods. The second dataset is the average reaction time (RT) and accuracy for repeated blocks of visual search trials.

#### Example 1: age and brain structure volume

Using data from (Raz et al., 2005) we assess the intra-individual relationship between age and cerebellar hemisphere brain (CBH) structural volume. Each measure was assessed on two occasions approximately 5 years apart, thus the data are longitudinal. The researchers found a negative association between age and CBH volume when using separate simple regression/correlation models for each of the two time periods (Raz et al., 2005).

Here, we demonstrate a variety of ways to analyze these data, using both simple regression/correlation and rmcorr: (a) separate simple regression/correlations, (b) rmcorr, and (c) simple regression/correlation using averaged data. For each of these three methods, we describe and plot the generated results and discuss their interpretation.

**Figure 5:**
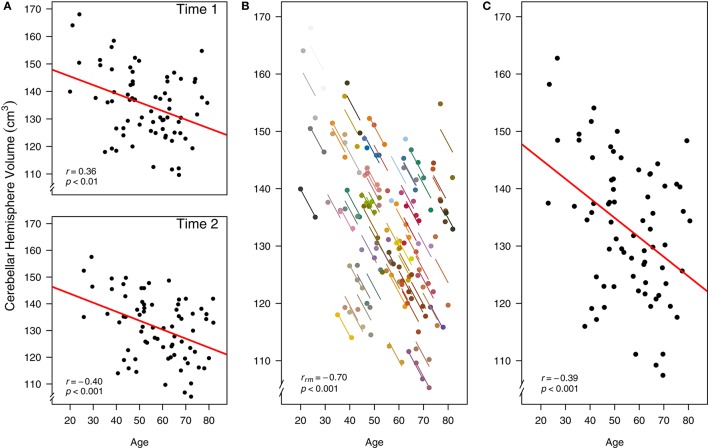
**Comparison of rmcorr and simple regression/correlation results for age and brain structure volume data**. Each dot represents one of two separate observations of age and CBH for a participant. **(A)** Separate simple regressions/correlations by time: each observation is treated as independent, represented by shading all the data points black. The red line is the fit of the simple regression/correlation. **(B)** Rmcorr: observations from the same participant are given the same color, with corresponding lines to show the rmcorr fit for each participant. **(C)** Simple regression/correlation: averaged by participant. Note that the effect size is greater (stronger negative relationship) using rmcorr **(B)** than with either use of simple regression models **(A)** and **(C)**. This figure was created using data from Raz et al. (2005).

First, we recreate the original cross-sectional (between-participants) analysis from the paper, where the relationship between age and CBH volume were assessed with separate simple regression/correlation models at Time 1 [*r*_(70)_ = −0.36, 95% CI [−0.54, −0.14], *p* < 0.01] and Time 2 [*r*_(70)_ = −0.40, 95% CI [−0.58, −0.19], *p* < 0.001; Figure 5A]. The interpretation of these results is cross-sectional: They indicate a moderately negative relationship between age and CBH volume across people, where older individuals tend to have a smaller volume and vice versa. If we instead analyze this data at the intra-individual level using rmcorr, we see a much stronger negative association between age and CBH volume, *r*_*rm*_ (71) = −0.70, 95% CI [−0.81, −0.56], *p* < 0.001 (Figure 5B). These results are interpreted longitudinally, and indicate that as an individual ages, CBH volume tends to decrease. Finally, it is possible to analyze the relationship between age and CBH volume using each participant's data averaged across the two time periods and a simple regression/correlation. This model produces similar results to the original cross-sectional analysis: *r*_(70)_ = −0.39, 95% CI [−0.57, −0.17], *p* < 0.01 (Figure 5C).

The three approaches address different research questions. The separate models analyze between-individual or cross-sectional change (Figure 5A), whereas rmcorr assesses the intra-individual or longitudinal change (Figure 5B). Taken together, differing magnitudes of associations indicate that the negative relationship for age and CBH volume is stronger within-individuals than between-individuals. Separate models presume that longitudinal and cross-sectional data are interchangeable, which is not the case here and is a general challenge with assessing the relationship between changes in age and brain volume.^5^ The third result assesses a similar question as the original, separate models (Figure 5C). Although this model is straightforward, using averaged data may reduce or obscure meaningful intra-individual variance, leading to decreased power.

Rmcorr results and the rmcorr plot (a simplified version of Figure 5B) are produced by running the following code:

1. Rmcorr: brainvolage.rmc < - rmcorr(participant = Participant, measure1 = Age, measure2 = Volume, dataset = raz2005)
2. Rmcorrplot: plot(brainvolage.rmc, raz2005, overall = F, lty = 2, xlab = “Age”, ylab = expression(Cerebellar~Hemisphere~Volume~(cmˆ{3})))

#### Example 2: visual search and response time

Using visual search data from one of the many search tasks reported in Gilden et al. (2010), we assess the intra-individual association between speed and accuracy. The continuous tradeoff between speed (reaction time) and accuracy (correct or incorrect) is well-known and occurs in a variety of tasks assessing cognitive processes (Wickelgren, 1977). In this experiment, 11 participants each completed four separate blocks of 288 visual search trials apiece. RT and accuracy were computed for each block, for each participant.

**Figure 6:**
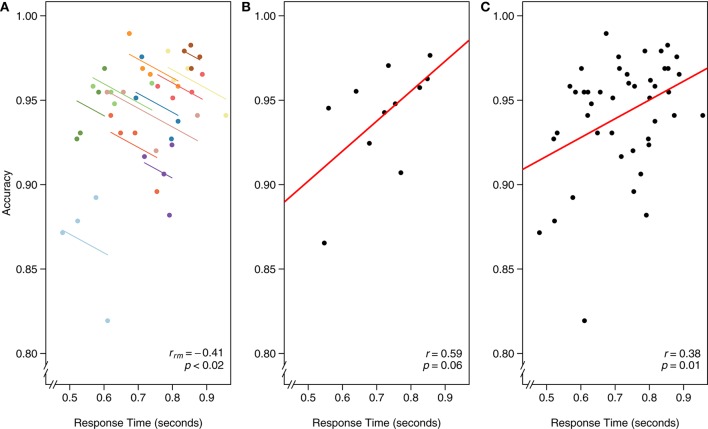
**The x-axis is reaction time (seconds) and the y-axis is accuracy in visual search. (A)** Rmcorr: each dot represents the average reaction time and accuracy for a block, color identifies participant, and colored lines show rmcorr fits for each participant. **(B)** Simple regression/correlation (averaged data): each dot represents a block, (improperly) treated as an independent observation. The red line is the fit to the simple regression/correlation. **(C)** Simple regression/correlation (aggregated data): improperly treating each dot as independent. This figure was created using data from Gilden et al. (2010).

As in the first example dataset, we worked through three different models for analyzing the relationship between RT and accuracy in Figure 6: (A) rmcorr, (B) simple regression/correlation (averaged data), and (C) simple regression/correlation (aggregated data): improperly treating each observation as independent. At the intra-individual level, rmcorr yields a negative relationship between speed and accuracy, *r*_*rm*_ (32) = −0.41, 95% CI [−0.66, −0.07], *p* < 0.02 (Figure 6A), consistent with a speed-accuracy tradeoff. This indicates that for a given individual, faster speed comes at the cost of reduced accuracy.

We can instead average each participant's RT and accuracy across the four experimental blocks and assess the inter-individual relationship between speed and accuracy. A simple regression/correlation model suggests a positive relationship, although the result is not significant: *r*_(9)_ = 0.59, 95% CI [−0.01, 0.88], *p* = 0.06 (Figure 6B). Note the decrease in power and that a large correlation, albeit a highly unstable one, is not significant because this model has only nine degrees of freedom. The first and second analyses appear contradictory. However, the appropriate analysis and interpretation of the results depend on the research question. If we want to quantify the speed-accuracy tradeoff, a phenomena that occurs within-individuals, the first analysis with rmcorr is appropriate. If we want to know, between participants and collapsed across blocks, if faster people tend to be more or less accurate, the second analysis is informative (though underpowered).

Finally, we show the result of aggregating all data and improperly treating each observation as independent. Because the data are not averaged, power is much higher, which may make this model initially attractive. Indeed, results show a significant positive relationship between RT and accuracy: *r*_(42)_ = 0.38, 95% CI [0.09, 0.61], *p* = 0.01 (Figure 6C). However, the model violates the assumption of independence; in essence, the data are treated as if 44 separate participants each completed one block of data. This incorrect specification overfits the model, making the results uninterpretable. We include this example to illustrate the importance of identifying the research question of interest, whether within-individuals, between-individuals, or both, and defining the analysis accordingly.

Rmcorr results and an rmcorr plot (similar to Figure 6A) are produced by running the following code:

1. Rmcorr: vissearch.rmc < - rmcorr(participant = sub, measure1 = rt, measure2 = acc, dataset = gilden2010)
2. Rmcorr plot: plot(vissearch.rmc, gilden2010, overall = F, lty = 2, xlab = “Reaction Time,” ylab = “Accuracy”)

## Conclusion

Unlike standard correlation/regression techniques, rmcorr can handle repeated measures data without violating independence assumptions or requiring first averaging the data. The strengths of rmcorr are in its potential for high statistical power, as well as its simplicity. Rmcorr is ideal for assessing a common association across individuals, specifically a homogenous intra-individual linear association relationship between two paired measures. The two examples provided above illustrate how rmcorr is straightforward to apply, visualize, and interpret with real data.

Because rmcorr analyzes paired repeated measures without averaging or violating the IID assumption it has clear advantages over simple regression/correlation. This is particularly true when there are violations of assumptions that result in biased and spurious parameter estimates. Researchers may find the analysis and visualization tools available in the **rmcorr** package useful for understanding and interpreting paired repeated measures data, especially in cases where these data exhibit non-intuitive patterns (e.g., Simpson's Paradox). This may include assessing and comparing the association within-individuals versus the association between individuals. For more complex datasets, rmcorr is not a replacement for multilevel modeling.

Future work will expand the examples and functionality of the **rmcorr** package. Rmcorr could complement multilevel modeling. For example, it may be informative for assessing collinearity in multilevel models and provide an effect size for a null multilevel model. Other possibilities include more detailed comparisons with a null multilevel model. Another future direction could be determining the stability of the rmcorr coefficient across different sample and effect sizes, building upon research simulating the stability of Pearson correlations (Schönbrodt and Perugini, 2013).

## Ethics statement

Both datasets are from previously published papers, no new data was collected for this manuscript.

## Author contributions

JB drafted the paper, LM wrote sections, and both revised the paper. Both authors contributed to the analyses and LM wrote the majority of the code for the R package. The authors approve the final version of the paper.

## Funding

This research was supported by the second author's appointment to the U.S. Army Research Laboratory Postdoctoral Fellowship Program administered by the Oak Ridge Associated Universities under Cooperative Agreement W911NF-16-2-0008. The views and conclusions contained in this document are those of the authors and should not be interpreted as representing the official policies, either expressed or implied, of the U.S. Army Research Laboratory or the U.S. government.

### Conflict of interest statement

The authors declare that the research was conducted in the absence of any commercial or financial relationships that could be construed as a potential conflict of interest.

## Footnotes

^1^In ANCOVA, parallel slopes are tested by adding an interaction term to the model for the factor by the covariate (e.g., Tabachnick and Fidell, 2007). A significant interaction indicates non-parallel slopes, which for ANCOVA may be considered an uninterpretable model depending on a variety of factors (e.g., Miller and Chapman, 2001; Tabachnick and Fidell, 2007). Although such an interaction test could be used with rmcorr, we contend it is not likely to be informative because non-parallel slopes would be appropriately indicated by the rmcorr effect size and multilevel modeling could be used instead.

^2^Slopes can be non-parallel in countless ways (e.g., strongly heterogeneous with opposing directions from positive to negative to weakly heterogeneous, all in the same direction with small variation). This is supported by evidence that, for ANCOVA, the degree of heterogeneity in slopes is what matters not merely the presence of a statistically significant interaction (Rogosa, 1980).

^3^Multilevel modeling has many different names (e.g., hierarchical linear modeling, generalized linear [mixed] modeling, and linear mixed effects modeling).

^4^In partial pooling the levels must influence each other, see Gelman and Hill (2007) and Kreft and de Leeuw (1998).

^5^Observed associations for changes in age and brain volume with longitudinal (within-individual) versus cross-sectional (between-individual) designs are not necessarily equivalent (Salthouse, 2011).
