# Corrigendum: Repeated Measures Correlation

**DOI:** 10.3389/fpsyg.2019.01201

**Published:** 2019-05-28

**Authors:** Jonathan Z. Bakdash, Laura R. Marusich

**Affiliations:** ^1^US Army Research Laboratory, Human Research and Engineering Directorate, Aberdeen Proving Ground, United States; ^2^US Army Laboratory South Field Element, Human Research and Engineering Directorate, University of Texas Arlington, Arlington, TX, United States

**Keywords:** correlation, repeated measures, individual differences, intra-individual, statistical power, multilevel modeling

In the original article, there were errors in Equations 2 and 3^1^. For Equations 2 and 3, *j* should be the participant and *i* the trial/repeated measure (*i* and *j* are swapped for consistency with Equation 1). In Equation 2, ${\bar{Measure2}}_{i}$ should be ${\bar{Measure1}}_{j}$. Additionally, the notation using *c* is confusing in Equation 2 and incorrect in Equation 3. Last, the left side of Equation 3 is incorrect, it should be the predicted value not its mean.

Corrections have been made to the **Background** section, subsection **Rmcorr and ANCOVA**, **Equations and rmcorr Table**, paragraphs four and five:

In Equations 2 and 3, Equation 1 is rewritten for rmcorr to show one measure as a function of its mean value, participant, and the covaried value of the other measure. Note following Equation 1, *i* and *j* are now exchanged for consistency: *j* = participant and *i* = trial or repeated measure.

(2) $$\begin{array}{r} {Measure1}_{ij}=\;{\bar{Measure1}}_{j}+\;Participant_{j}+\;\beta \left({Measure2}_{ij}-\;{\bar{Measure2}}_{j}\right)+\;\varepsilon_{ij} \end{array}$$

*Measure*1 and *Measure*2 are exchangeable.

*Measure*1_*ij*_ is the value of *Measure*1 for the *j*th participant at their *i*th trial.

${\bar{Measure1}}_{j}$ is the mean of *Measure*1 (all *i* trials) for the *j*th participant.

*Participant*_*j*_ is a unique identifier that acts as a dummy or proxy coded variable.

β is the value of the covariate, which is the overall or common slope.

*Measure*2_*ij*_ is the value of *Measure*2 for the *j*th participant at their *i*th trial.

${\bar{Measure2}}_{j}$ is the mean of *Measure*2 (all *i* trials) for the *j*th participant.

ε_*ij*_ is the error for the *j*th participant at their *i*th trial.

Equation 2 is rewritten to calculate the predicted value of the rmcorr regression line for each participant by trial. We drop the error term because we do not fit a confidence interval for the regression line.

(3) $$\begin{array}{r} {Measure1}_{ij}^{\prime }=\;{\bar{Measure1}}_{j}+\;Participant_{j}+\;\beta \left({Measure2}_{ij}-\;{\bar{Measure2}}_{j}\right) \end{array}$$

$Measure1_{ij}{}^{\prime }$ is the predicted y-value of *Measure*1 for the *j*th participant at their *i*th trial.

*Measure*2_*ij*_ is the actual x-value which corresponds to the predicted y-value in the regression line.

Please note that the **rmcorr package** has always produced the corrected results.

The authors apologize for these errors and state that they do not change the scientific conclusions of the article in any way. The original article has been updated.

## Footnotes

^1^We are grateful to Alexander Wedel for identifying these errors and also thank him for helping correct the equations.

