# Supplementary material for: Repeated Measures Correlation
Source: Front Psychol. 2017 Apr 7;8:456. doi: 10.3389/fpsyg.2017.00456 (PMC5383908; doi:10.3389/fpsyg.2017.00456)
Supplement: Supplementary file 1 [file DataSheet1.pdf]

1 Appendix A: Additional R Packages Used in Paper

The following is a list of citations for R packages, not cited in References. These packages were used to create figures in this paper that are reproducible with the corresponding R Markdown file.

Allaire, J. J., Horner, J., Marti, V., & Porte, N. (2015). markdown: 'Markdown' Rendering for R. R package version 0.7.7. https://CRAN.R-project.org/package=markdown

Bates, D., Maechler, N., Bolker, B., and Walker, S. (2015). Fitting Linear Mixed-Effects Models Using lme4. *Journal of Statistical Software*, *67*(1), 1-48. doi:10.18637/jss.v067.i01.

Champely, S. (2015). pwr: Basic Functions for Power Analysis. R package version 1.1-3. https://CRAN.R-project.org/package=pwr.

Harrell Jr, F. E. with contributions from C. Dupont and many others. (2015). Hmisc: Harrell Miscellaneous. R package version 3.17-1. https://CRAN.R-project.org/package=Hmisc.

Knowles, J. E., & Frederick, C. (2016). merTools: Tools for Analyzing Mixed Effect Regression Models. R package version 0.3.0. https://CRAN.R-project.org/package=merTools

Lemon, J. (2006) Plotrix: A package in the red light district of R. *R-News, 6*(4): 8-12.

Mazerolle, M. J. (2016) AICcmodavg: Model selection and multimodel inference based on (Q)AIC(c). R package version 2.1-0. https://cran.r-project.org/package=AICcmodavg

Neuwirth, E. (2014). RColorBrewer: ColorBrewer Palettes. R package version 1.1-2. https://CRAN.R-project.org/package=RColorBrewer.

Revelle, W. (2015) psych: Procedures for Personality and Psychological Research, Northwestern University, Evanston, Illinois, USA, http://CRAN.R-project.org/package=psych Version = 1.5.8.

Rinker, T. W. & Kurkiewicz, D. (2015). pacman: Package Management for R. version 0.4.1. University at Buffalo. Buffalo, New York. http://github.com/trinker/pacman

Wickham, H. and Chang, W. (2015). devtools: Tools to Make Developing R Packages Easier. R package version 1.9.1. https://CRAN.R-project.org/package=devtools.

Wickham, H. (2009). *ggplot2: Elegant Graphics for Data Analysis*. New York: Springer-Verlag.

Wright, K. (2016). pals: Color Palettes, Colormaps, and Tools to Evaluate Them. R package version 1.0. https://CRAN.R-project.org/package=pals

Xie, Y. (2016). knitr: A General-Purpose Package for Dynamic Report Generation in R. R package version 1.13.

Xie, Y. (2015). *Dynamic Documents with R and knitr*. 2nd Edition. New York: Chapman and Hall/CRC.

2 Appendix B: Proof for Degrees of Freedom Approximation

The degrees of freedom for rmcorr are approximately $X \approx (k-1)$ times greater than the degrees of freedom for the Pearson correlation. To prove this approximation, first, the degrees of freedom for the Pearson correlation coefficient $(N-2)$ are multiplied by an unknown variable (X). Then this side of the equation is set equal to the rmcorr error degrees of freedom (Equation 1). Next, X is solved (Equation 2). Then, the numerator and denominator of the right side of the equation are divided by N (Equation 3) and simplified (Equation 4). Limits are used to prove convergence between the exact formula (Equation 5) and the approximation (Equation 6). Last, as N is large, the degrees of freedom for rmcorr can be further simplified (Equation 7) to provide the effective sample size for power analysis with a Pearson correlation.

$$\boldsymbol{X \times (N-2) = N(k-1) - 1} \tag{1}$$

$$\boldsymbol{X = \frac{N(k-1)-1}{N-2}} \tag{2}$$

$$\boldsymbol{X = \frac{\frac{N(k-1)-1}{N}}{\frac{(N-2)}{N}}} \tag{3}$$

$$\boldsymbol{X = \frac{k-1-\frac{1}{N}}{1-\frac{2}{N}}} \tag{4}$$

As N goes to infinity, the limit of $\boldsymbol{X \approx (k-1)}$.

$$\boldsymbol{\lim_{N\to\infty} \frac{k-1-\frac{1}{N}}{1-\frac{2}{N}} = k-1} \tag{5}$$

$$\boldsymbol{X \approx (k-1)} \tag{6}$$

3 Appendix C: Rmcorr and Multilevel Modeling

Rmcorr and multilevel modeling are directly compared using the two example datasets previously described. The graphical comparisons illustrate rmcorr is approximately equivalent to a null multilevel model (varying intercepts for each individual and a fixed/common slope). It is critical to note the comparability between rmcorr and multilevel modeling only holds for a common slope. If slopes meaningfully vary among individuals, especially in direction, rmcorr results are likely to produce an effect size around zero. In such a case, a multilevel model that includes varying effects for slope is called for if such a model is supported by theory, sufficient data, and/or model fit statistics.

Example 1: Brain Volume and Age

There are only two paired estimates per participant in the brain volume and age data. This is insufficient data to fit a multilevel model with a varying slope for each individual. **Figure 1** shows rmcorr and a multilevel model with a varying intercept and fixed slope.

Appendix C Figure 1

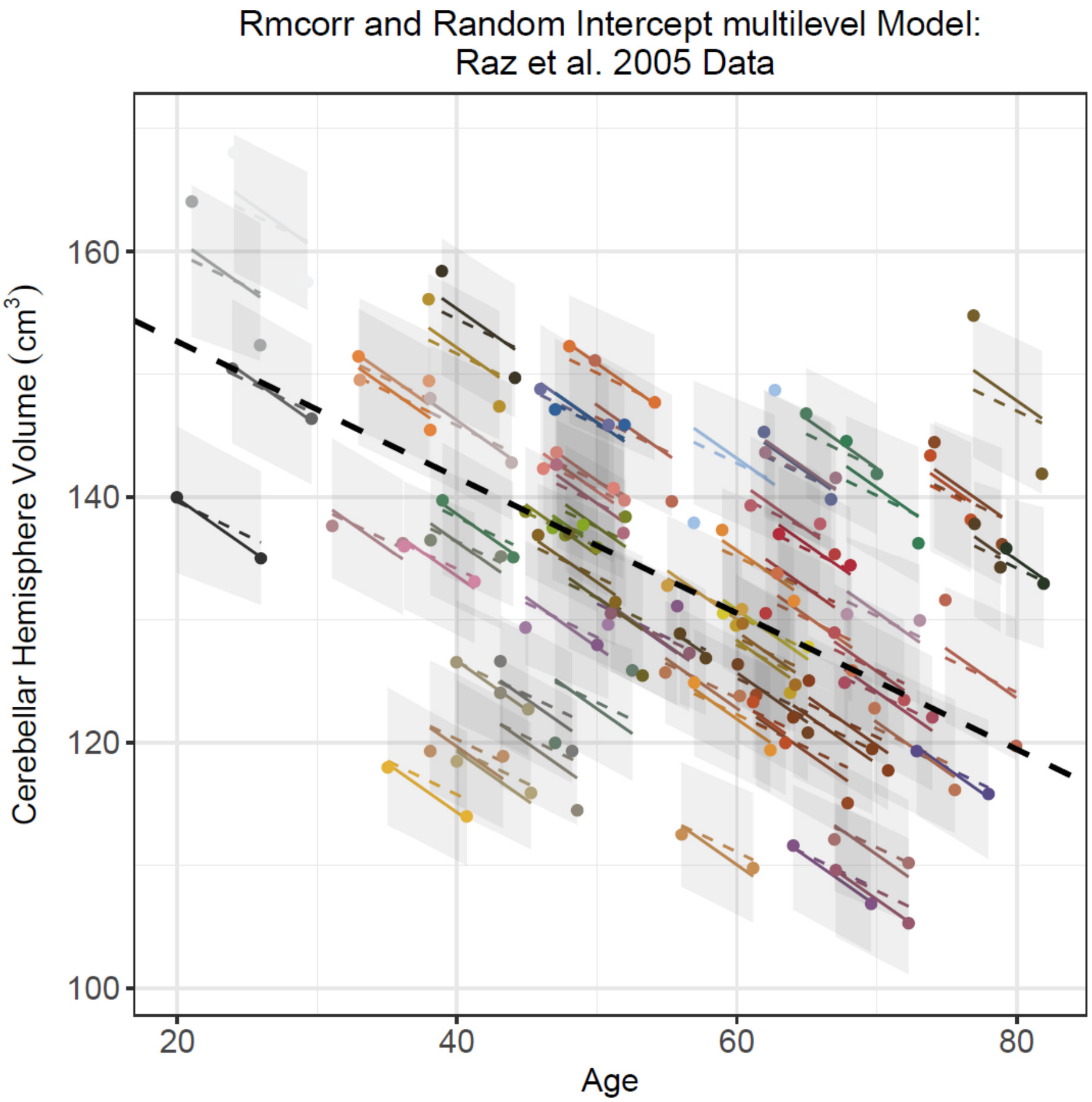

Appendix C, Figure 1: Dots are actual data values, with color indicating participant. Solid colored lines show the rmcorr model fit. The multilevel model fit is indicated by the dashed colored lines for Level 1 (participant) effects and the dashed black line for Level 2 (experiment) effects. The shaded areas are 95% confidence intervals for Level 1 effects. Note the models clearly overlap, despite the absence of confidence intervals for rmcorr.

Example 2: Visual Search

The visual search data have four paired estimates per participant, which is just below the guideline of five to eight data points per participants to fit a varying slope (Bolker, 2015). Nevertheless, we fit two multilevel models: 1) Null model with a varying intercept and fixed slope and 2) A more complex model with a varying intercept and varying slope. Using model

comparison with Akaike's Information Criterion corrected, the null model was 13.43 times more likely than the more complex model. Moreover, the random slope model exhibits convergence problems for calculating confidence intervals. This "simple" comparison between two multilevel models demonstrates overfitting and misspecification (Bates et al., 2015; Matuschek et al., 2015). **Figure 2** shows rmcorr and a null multilevel model with a varying intercept and fixed slope.

Appendix C Figure 2

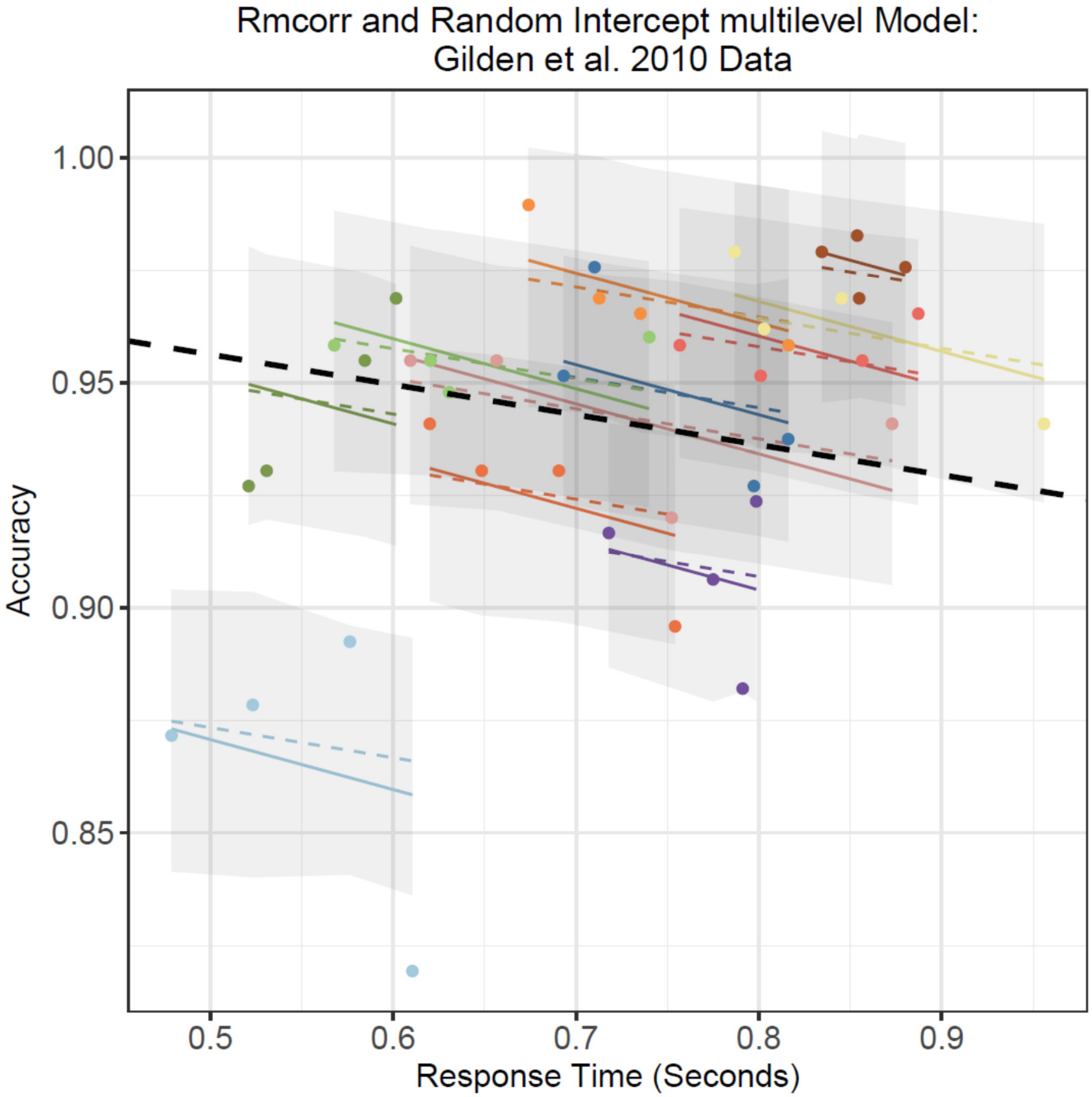

Appendix C, Figure 2: Dots are actual data values, with color indicating participant. Solid colored lines show the rmcorr model fit. The multilevel model fit is indicated by the dashed colored lines for Level 1 (participant) effects and the dashed black line for Level 2 (experiment) effects. The shaded areas are 95% confidence intervals for Level 1 effects. Note the models clearly overlap, despite the absence of confidence intervals for rmcorr.
